# Spatiotemporal analysis of multi-scale cell structure in spheroid culture reveals hypertrophic chondrocyte differentiation

**DOI:** 10.1007/s00441-024-03905-7

**Published:** 2024-07-23

**Authors:** Kosei Tomida, Jeonghyun Kim, Eijiro Maeda, Taiji Adachi, Takeo Matsumoto

**Affiliations:** 1 Department of Mechanical Systems Engineering, Graduate School of Engineering, Nagoya University, Nagoya, Japan; 2 Department of Biosystems Science, Institute for Life and Medical Sciences, Kyoto University, Kyoto, Japan

**Keywords:** 3D image analysis, Chromatin condensation, Nucleus, Hypertrophic chondrocyte, ATDC5, Spheroid

## Abstract

3D cell culture has emerged as a promising approach to replicate the complex behaviors of cells within living organisms. This study aims to analyze spatiotemporal behavior of the morphological characteristics of cell structure at multiscale in 3D scaffold-free spheroids using chondrogenic progenitor ATDC5 cells. Over a 14-day culture period, it exhibited cell hypertrophy in the spheroids regarding cellular and nuclear size as well as changes in morphology. Moreover, biological analysis indicated a signification up-regulation of normal chondrocyte as well as hypertrophic chondrocyte markers, suggesting early hypertrophic chondrocyte differentiation. Cell nuclei underwent changes in volume, sphericity, and distribution in spheroid over time, indicating alterations in chromatin organization. The ratio of chromatin condensation volume to cell nuclear volume decreased as the cell nuclei enlarged, potentially signifying changes in chromatin state during hypertrophic chondrocyte differentiation. Our image analysis techniques in this present study enabled detailed morphological measurement of cell structure at multi-scale, which can be applied to various 3D culture models for in-depth investigation.

**Supplementary Information:**

The online version contains supplementary material available at 10.1007/s00441-024-03905-7.

## Introduction

Endochondral ossification is a process of long bone formation during embryonic development, which begins with the condensation of mesenchymal cells, followed by chondrogenic differentiation, and the replacement of mineralized cartilage tissue by bone tissue (Gibson et al. 1995; Long and Ornitz 2013). During the process, prechondrogenic cells undergo a series of differentiation in the order of proliferating chondrocytes, chondrocytes, and hypertrophic chondrocytes, while showing remarkable alterations in functions and morphology (Kronenberg 2003). Particularly, the hypertrophic chondrocyte differentiation involves multiple essential events, which include the enlargement in the size for elongation of bone, changes in their genetic program to synthesize Type X collagen for further mineral deposition and apoptosis to provide the material and space for bone formation (Kronenberg 2003). Molecular mechanisms during the hypertrophic chondrocyte differentiation have been extensively studied using primary chondrocytes and a chondrogenic cell line in a two-dimensional (2D) culture over the past several decades (Lewis et al. 2011; Studer et al. 2012; Yao and Wang 2013). On the other hand, more attention is being paid to the physiology in a three-dimensional (3D) culture because the chondrocytes rapidly dedifferentiate on a 2D substrate culture condition (Darling and Athanasiou 2005; Furukawa et al. 2003). Several groups have reported chondrogenesis-induced pellet models (about several millimeters in diameter), which resulted in the cells showing hypertrophic chondrocyte differentiation and cartilage-like protein synthesis (Kimura et al. 2018; Pretemer et al. 2021; Scotti et al. 2013). In addition, our group previously reported a scaffold-free 3D spheroid model with about 100 – 200 µm, representing greater gene expressions of hypertrophic chondrocyte markers beyond the 2D culture (Kim et al. 2022; Kim and Adachi 2021).

While biochemical assays and histochemistry have been practical methods to characterize the physiological state of cells, morphological analysis also would provide us comprehensive cues to infer cellular states. Numerous literatures reported the biological significance of cellular and nuclear morphology as they indicate the mechanical state that influences downstream functionality such as chromatin conformation and, in turn, transcriptional activities (Amar et al. 2021; Kalukula et al. 2022). Thus, the morphological analysis is a promising approach for further insights into the understanding of hypertrophic chondrocyte differentiation.

Pretemer et al. observed cellular morphology using tissue slices of chondrogenesis-induced pellet model and found that hypertrophic chondrocyte differentiation begins at the periphery and later progresses in the core, with concomitant alteration in cellular morphology (Pretemer et al. 2021). This study raised further questions about the three-dimensional morphology of the nucleus and chromatin during hypertrophic chondrocyte differentiation because it has been assured that the reorganization of chromatin structure via deformation of the cell nucleus may affect cellular function (Amar et al. 2021). The 3D observation is also preferable in terms of analyzing nuclear and chromatin morphology because tissue slices might lack the 3D information. However, 3D imaging with confocal laser scan microscopy in the pellet models was practically challenging due to its size exceeding the working distance of the objective lens. Therefore, imaging the whole structure of the pellet in high magnification was difficult and this hindered us from capturing the spatial feature within the model. We previously reported a spheroid model with 100 – 200 µm diameter, reconstructed with chondrogenic progenitor cells ATDC5, which exhibited early hypertrophic chondrocyte differentiation. The whole body of the spheroid model can be captured within the working distance of confocal laser scan microscopy.

In this study, we aim to induce hypertrophic chondrocyte differentiation of chondrogenic progenitor cells in spheroid culture and carry out 3D imaging analysis using confocal laser scan microscopy. Furthermore, we quantitatively analyze the 3D morphological features and the distribution of nuclei and chromatin condensations in the spheroid.

## Materials and methods

### Cell culture and fabrication of spheroids

Mouse chondrogenic progenitor cell line ATDC5 (RIKEN Bioresource Center, Japan) was cultured in DMEM/F-12 (Gibco, USA) containing 5% fetal bovine serum (Gibco, USA) and 1% penicillin-streptomycin (Nacalai Tesque, Japan) in a humidified incubator (37℃, 5% CO_2_). Cell passage was conducted every 3-4 days before reaching 80% – 90% confluence. Spheroids were prepared by subculturing in a U-bottom ultra-low attachment 96-well plate (Thermo Fisher Scientific, USA) following previously reported methods (Kim et al. 2020, 2022). 2,500 cells were seeded to each well and incubated for 2, 4, 7, and 14 days, with medium changes every 2 – 3 days.

### Real-time PCR

Upon the sample collection at day 2 and 14 of culture, spheroids were lysed using Isogen II (Nippon Gene, Japan), and total RNA was extracted using RNA Mini Kit (Invitrogen, USA). Immediately thereafter, cDNA was synthesized using a DNA Reverse Transcription Kit (Toyobo, Japan). cDNA was mixed with PowerUp SYBR Green Master Mix (Thermo Fisher Scientific, USA), and PCR reactions were performed using QuantStudio1 (Thermo Fisher Scientific, USA). All mRNA expression levels were normalized against the housekeeping gene glyceraldehyde-3-phosphate dehydrogenase (*Gapdh*). To examine the chondrocyte differentiation marker, mRNA expression levels of aggrecan (*Acan*) and collagen type II α1 chain (*Col2a1*) were quantified. The mRNA expression levels of fibroblast growth factor receptor 3 (*Fgfr3*), collagen type X α1 chain (*Col10a1*), and matrix metallopeptidase (*Mmp13*) were examined to evaluate hypertrophic chondrocyte marker.

Relative mRNA expression values were calculated using the 2^-ΔΔCt^ method. ΔΔCt values were normalized against a mean value of the day 2 spheroids for each gene. The primer sets used in this study are listed in Supplementary Table S1.

### Immunofluorescence staining and imaging

Spheroids were collected at day 2, 7, and 14 of culture for immunofluorescence staining experiments, rinsed in phosphate-buffered saline (PBS), and then fixed in 10% formaldehyde neutral buffer solution at room temperature (RT) for 2 h. After permeabilization using 0.5% Triton X‐100 (Sigma Aldrich, USA) for 15 min, the samples were blocked with 4% bovine serum albumin (Sigma Aldrich, USA) for 1 h. Then, anti-collagen type X primary antibody (Abcam, UK) diluted in PBS (1:200) was added and incubated at 4°C overnight to analyze the hypertrophic chondrocyte marker (Type X collagen (COL10)). After washing with PBS, the samples were treated with Alexa Fluor 488 secondary antibody (Invitrogen, USA) in PBS (1:200), and Alexa Fluor 546 phalloidin (Invitrogen, USA) in PBS (1:200) for 2 h at RT to visualize COL10 (green) and actin filaments (red), respectively. Hoechst 33342 (Invitrogen, USA) in PBS (1:500) was added for 1 h at RT to stain the nuclei (blue). After the staining, samples were washed with PBS three times. Before observation, the samples were immersed in a 60% aqueous iodixanol solution (Serumwerk, Germany) for 1 h to match the refractive index of the solution and that of the samples for optical clearing as previously reported (Boothe et al. 2017; Inagaki et al. 2023; Maeda et al. 2022). The samples were subsequently observed using an LSM880 with a 63x/1.4 NA oil objective lens. A 3D image of the hemispherical portion of the spheroid was acquired at a depth that fell within the working distance of the lens (0.19 mm) because the diameter of some spheroids exceeded the working distance of the objective lens. Negative controls without primary antibodies were used for background correction for imaging COL10.

### Cryosections and fluorescence staining

The spheroids were washed in PBS, fixed in 10% formaldehyde neutral buffer solution for 2 h at RT, immersed in 30% sucrose, and then embedded in optimal cutting temperature compound (Sakura FineTek, Japan) to prepare a frozen block. The sections were cut in 10 μm using a cryostat (Leica, Germany). The sections were washed with water to remove the compound. The sections were then stained with Alexa Fluor 546 phalloidin (1:200) for 1 h and Hoechst 33342 (1:500) for 30 min at RT. The staining images were observed using LSM880 (Zeiss, Germany) with a 63x/1.4 NA Oil objective lens. All the 2D images were analyzed using ImageJ software (NIH, USA). The circularity is calculated using a built-in function of ImageJ:

1 $$\text{Cell circularity}\,=\,\frac{4\pi A}{{P}^{2}}$$

where $A$ and $P$ are the area of the cell (contour of actin cortex) and perimeter of the cell, respectively.

### 3D image analysis on nuclei

Each nucleus and chromatin condensation were manually segmented using Imaris (Bitplane, UK). To analyze the three-dimensional nuclear distribution, we performed the segmentation for all nuclei within one-fourth of the spheroid hemisphere. The nuclear sphericity was calculated using a built-in function of Imaris as:

2 $$\mathrm{The\,nuclear\,sphericity}\,=\,\frac{{\pi }^{1/3}{\left({6V}_{n}\right)}^{2/3}}{{A}_{N}}$$

where ${V}_{n}$ and ${A}_{N}$ are the volume and the surface area of the nucleus, respectively (the sphericity becomes 1 if the nucleus is sphere shape). For the analysis of chromatin condensation, we selected nuclei that possess distinct chromatin condensations in Fig. 5(a). The total number and volume of chromatin condensations in each nucleus were examined by measuring relative nuclear position from the center of the spheroid because each spheroids had different diameters.

### Statistics analysis

All statistical analyses were performed using the statistical software R Studio. The Shapiro-Wilk test was used to check if data is normally distributed before proceeding with statistics for all the experiments. The non-parametric Friedman test was used to analyze the projected area of spheroids between groups with different incubation days. If the statistical significance was confirmed, the Steel-Dwass test was used as a post-hoc test. The differences in gene expression between the two groups with different incubation days were examined using the Mann-Whitney U test. The Kruskal Wallis test was used to test the difference in cell cross-sectional area, roundness, cell nucleus volume, cell nucleus sphericity, total volume of chromatin condensations, and number of chromatin condensations with different culture days, followed by Steel-Dwass multiple comparison test if statistical significance was confirmed. In the correlation analysis, the correlation between two different variables was analyzed by Spearman's rank correlation coefficient. *P* values less than 0.05 were considered statistically significant in all analyses.

## Results

### The ATDC5 spheroid hypertrophied and exhibited an alteration in cell distribution during 14 days of culture

**Fig. 1:**
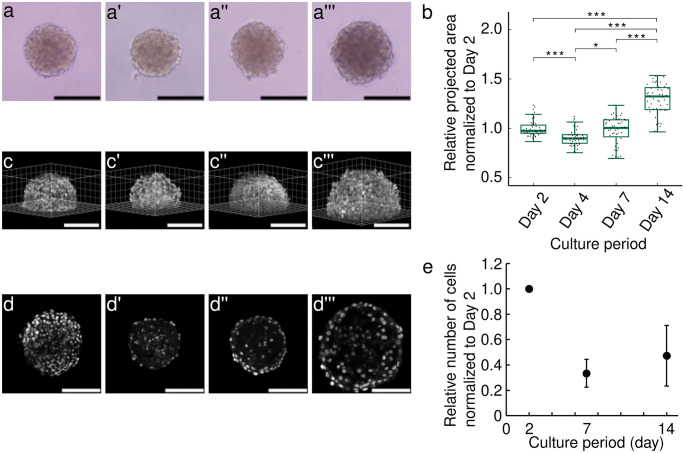
Temporal change of the spheroid morphology. Bright-field images of spheroids after **a** 2-day, **a’** 4-day, **a’’** 7-day, and **a’’’** 14-day incubation after fabrication. Black bars indicate 100 µm. **b** Boxplot of the relative projected area of spheroids at day 4, 7, and 14 normalized to day 2 (n = 47 from four independent experiments; p value was calculated from Friedman test followed by Steel-Dwass post-hoc test; *p < 0.05, **p < 0.01, ***p < 0.001). 3D images of hemispherical spheroids with nuclei stained by Hoechst 33,342 on **c** 2-day, **c’** 4-day, **c’’** 7-day, and **c’’’** 14-day incubation after fabrication. White bars indicate 100 µm. Cross-sectional images of the nuclei in spheroids at **d** day 2, **d’** day 4, **d’’** day 7, and **d’’’** day 14. White bars indicate 100 µm. **e** The relative number of nuclei in the hemispherical spheroid at day 2, 7, and 14. The number of nuclei on day 2, 7, and 14 was calculated relative to that of day 2. The bars represent the mean ± standard deviation (n = 3 from three independent experiments)

Figures 1(a–a’’’) represent bright-field images of ATDC5 spheroid incubated for 2, 4, 7, and 14 days. The projected area decreased significantly from day 2 to day 4 (*p* < 0.001 for day 2 vs day 4), and then increased gradually up to day 14 as shown in Fig. 1(b) (*p* < 0.001 for day 4 vs day 14). The 3D hemispherical spheroid in Fig. 1(c–c’’’) shows the hypertrophied spheroid. In addition, the distribution of the nuclei within the spheroid became sparse over 14 days as shown in Fig. 1(d–d’’’). Particularly, the cell density appeared to decrease at day 4 compared to day 2. Figure 1(e) shows that the number of the cell nuclei in the spheroid decreased to about 30% from day 2 to day 7 and then increased from day 7 to day 14.

### ATDC5 spheroid culture for 14 days induced the early stage of hypertrophic chondrocyte differentiation

**Fig. 2:**
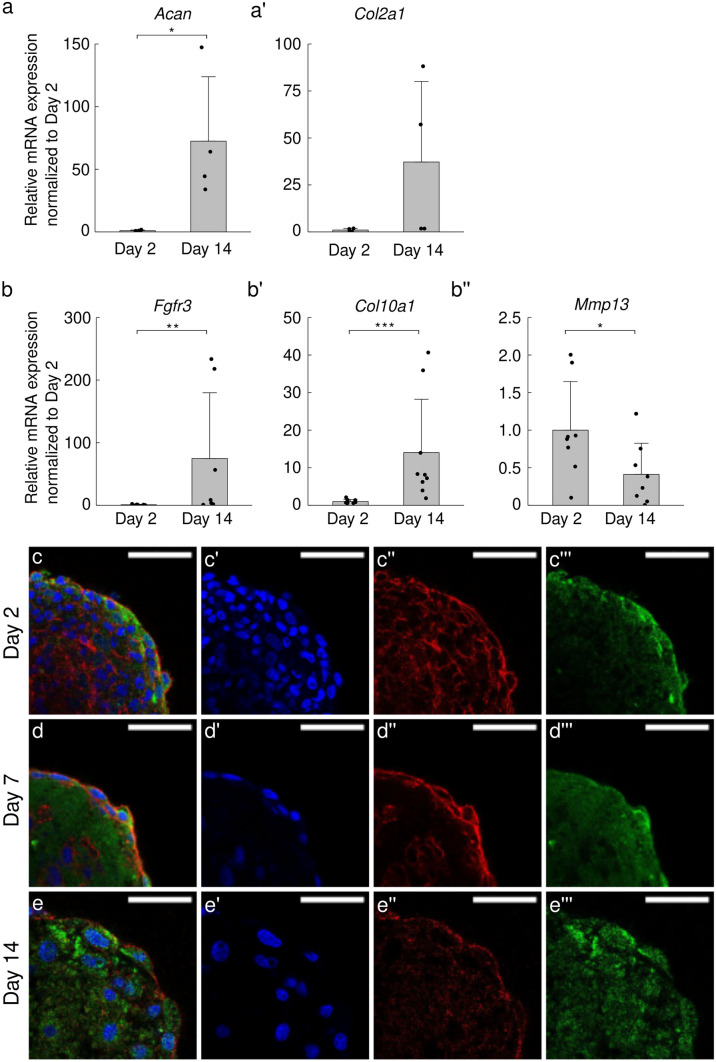
Chondrogenesis in the ATDC5 spheroids after 14 days of cultivation. The mRNA expressions of chondrocyte differentiation markers (**a** Acan and **a’** Col2a1) normalized to the mean value of day 2. The mRNA expressions of hypertrophic chondrocyte differentiation markers (**b** Fgfr3, **b’** Col10a1, and **b’’** Mmp13) normalized to the mean value of day 2. Bars represent the mean ± standard deviation obtained from three independent experiments (p value was calculated from Mann-Whitney U-test; *p < 0.05, **p < 0.01, ***p < 0.001). Immunofluorescence images of the cryosectioned samples for the spheroids on **c** day 2, **d** day 7, and **e** day 14, were stained for nucleus (blue;’), actin filaments (red;’’), and type X collagen (COL10;’’’) (green), respectively. White bars indicate 50 µm

Spheroids at day 2 and 14 were collected to evaluate gene expression of chondrocyte marker and hypertrophic chondrocyte marker. Figure 2(a–a’) show the gene expression of chondrocyte markers at day 2 and 14, where expression levels are normalized by those of day 2 for each gene. *Acan* was significantly increased (72.4-fold change, *p* < 0.05) at day 14 and *Col2a1* was also upregulated (37.2-fold change, *p* = 0.114) at day 14. Figure 2(b–b’’) represent the gene expression level of early and late hypertrophic chondrocyte marker genes. The early hypertrophic chondrocyte markers *Fgfr3* and *Col10a1* were significantly upregulated at day 14 (74.7-fold change (*p* < 0.001) and 14.0-fold change (*p* < 0.001), respectively). On the other hand, late hypertrophic chondrocyte marker *Mmp13* exhibited a significant decrease at day 14 (0.412-fold change, *p* < 0.05).

Figure 2(c–e’’’) shows fluorescent staining images of the middle plane of spheroids (blue: nucleus, red: actin filament, green: type X collagen (COL10)) at day 2, 7, and 14. Notably, the distribution and the size of nuclei were altered from day 2 to 14. The number of nuclei in the hemispherical spheroid decreased from day 2 to 7 and increased at 14 as shown in Fig. 1(e’). We also examined the cell viability in the spheroid at day 2 and 14 in Fig. S1. At day 2, a large number of cells presented cell death particularly inside of the spheroid. Many of the dead cells disappeared and the cells around the spheroid periphery remained alive at day 14. Moreover, Fig. 2 (c’’’) revealed that the COL10 expression was mainly detected at the surface area of the spheroid on day 2, whereas the COL10 expression was detected entirely in the spheroids at (d’’’) day 7 and (e’’’)14.

### ATDC5 spheroid culture induced cellular hypertrophy and increased the cellular circularity over 14 days of culture

**Fig. 3:**
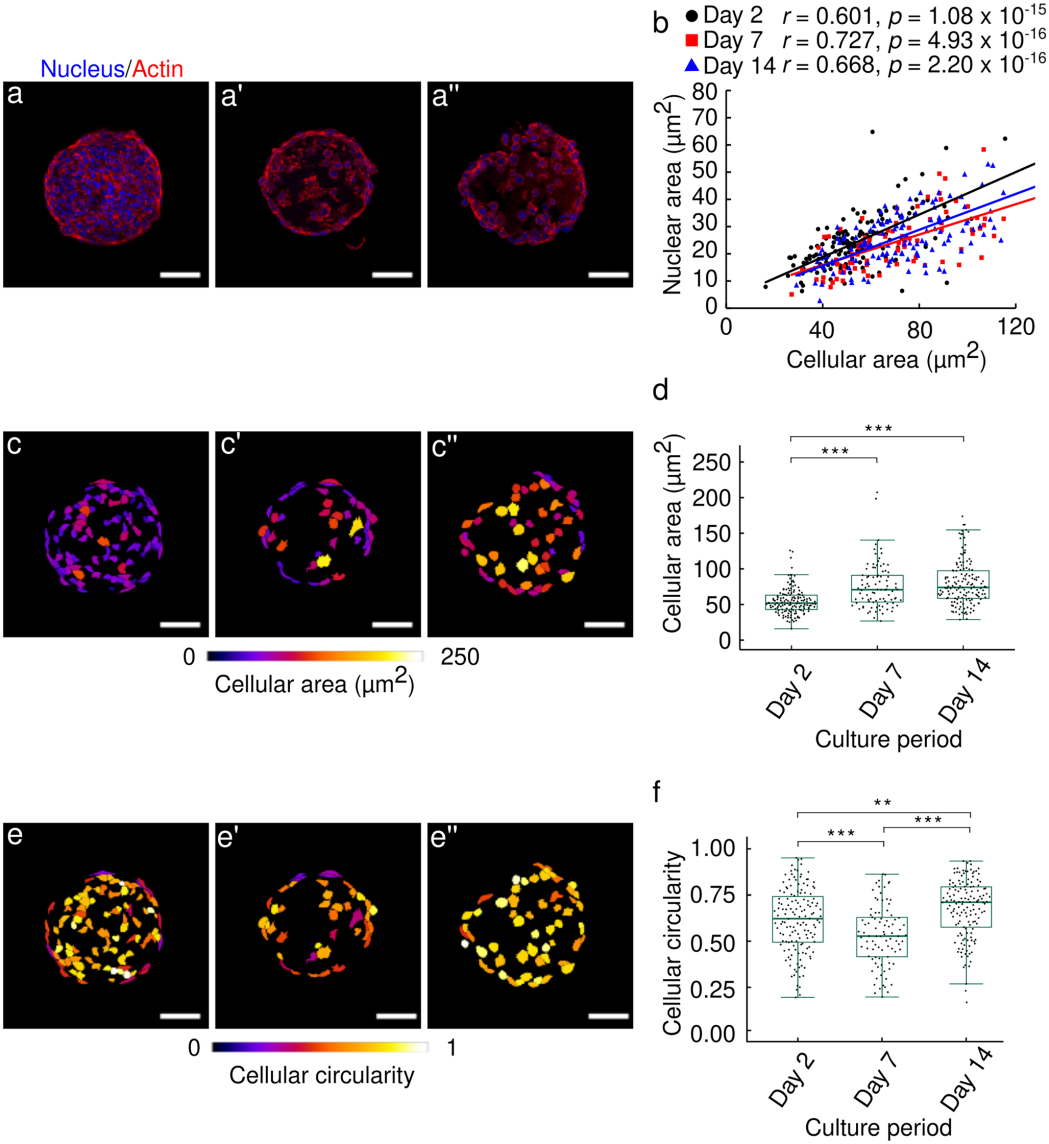
Two-dimensional image analysis for cellular morphologies. Fluorescence images of nuclei (blue) and actin filaments (red) in the spheroid at **a** day 2, **a’** day 7, and **a’’** day 14. **b** Relationship between the cellular area and nuclear area in the cross-sectional images of the spheroids at day 2, 7, and 14. The correlation between the cellular area and nuclear area was examined by Spearman’s correlation test. Representative images depicting the cellular area for the spheroids at **c** day 2, **c’** day 7, and **c’’** day 14. Colors filling in each cell indicate cellular area corresponding to the color bar. **d** Boxplot of the cellular area for the spheroids at day 2, 7, and 14. Bars represent the mean ± standard deviation obtained from three independent experiments (p value was calculated from Kruskal-Wallis test followed by Steel-Dwass post hoc test; *p < 0.05, **p < 0.01, ***p < 0.001). Representative images depicting the cellular circularity in the spheroid at **e** day 2, **e’** day 7, and **e’’** day 14. Colors filling in each cell indicate cellular circularity corresponding to the color bar. **f** Boxplot of the cellular circularity for the spheroids at day 2, 7, and 14. Bars represent the mean ± standard deviation obtained from three independent experiments (n, number of cells; n = 146 for day 2; n = 90 for day 7; n = 145 for day 14; p value was calculated from Kruskal-Wallis test followed by Steel-Dwass post hoc test; *p < 0.05, **p < 0.01, ***p < 0.001). White bars in each image indicate 50 µm

We observed the F-actin using cryosections of spheroids to obtain clear signals in Fig. 3(a–a’’). We confirmed that the nuclear and cellular size have a positive correlation as shown in Fig. 3(b). Moreover, cellular area significantly increased at day 7 and 14 compared to day 2 (*p* < 0.001, for day 2 vs day 7 and day 2 vs day 14) as shown in Fig. 3(c–c’’) and (d). In addition, the cellular circularity analysis in Fig. 3(e–e’’) revealed that the cells at the surface of the spheroid appeared to have a relatively elongated morphology compared to inner cells at day 2, 7, and 14. The cellular circularity significantly decreased from day 2 to 7 (*p* < 0.001) and then increased from day 7 to 14 (*p* < 0.001) as shown in Fig. 3(f).

### Cell nuclear morphology and their spatial distribution were altered after 14 days of spheroid culture

**Fig. 4:**
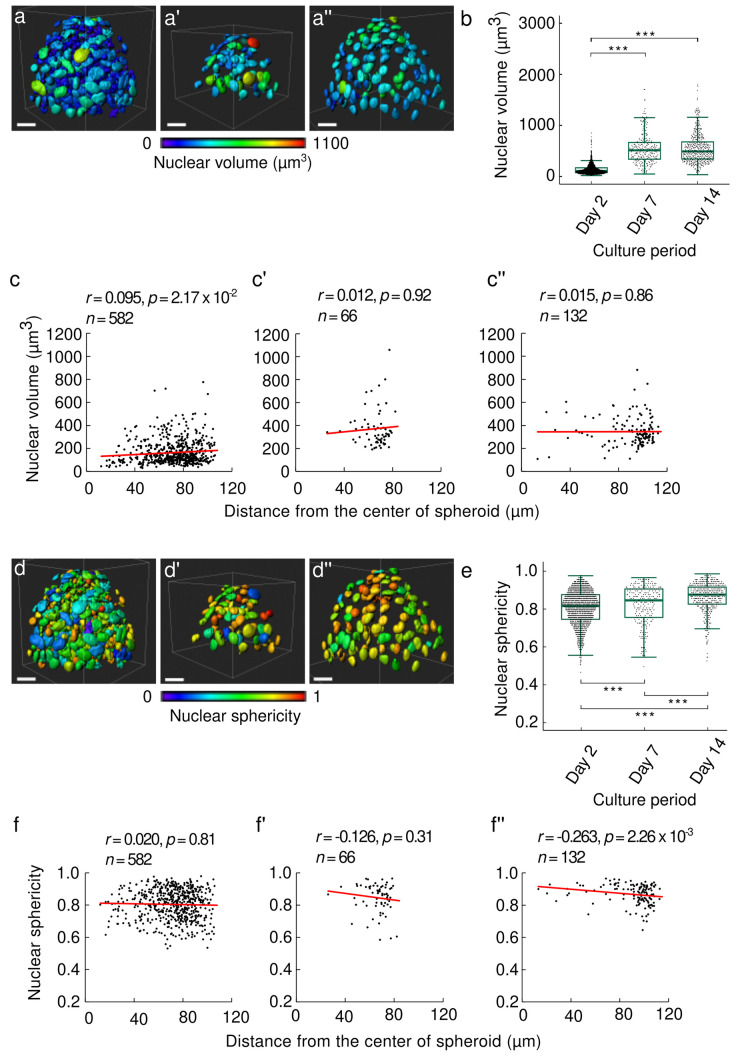
Three-dimensional (3D) spatiotemporal image analysis for nuclear morphologies in ATDC5 spheroids. Representative images of the 3D image analysis for the nuclear volume of the spheroids at **a** day 2, **a’** day 7, and **a’’** day 14. The color in each nucleus indicates its volume corresponding to the color bar. **b** Boxplot of nuclear volume for the spheroids at day 2, 7, and 14. n indicates the number of cells. Representative result of the relationship between the nuclear position and nuclear volume at **c** day 2, **c’** day 7, and **c’’** day 14. The correlation between the nuclear position and the nuclear volume was examined by Spearman’s correlation test. Representative images of the 3D image analysis for nuclear sphericity of spheroid at **d** day 2, **d’** day 7, and **d’’** day 14. The color in each nucleus indicates its sphericity corresponding to the color bar. **e** Boxplot of the nuclear sphericity for the spheroids at day 2, 7, and 14. Relationship between the distance from the center of the spheroid for each nucleus and nuclear sphericity at **f** day 2, **f’** day 7, and **f’’** day 14. The correlation between the nuclear position and the nuclear sphericity was examined by Spearman’s correlation test (n, number of cells; p value was calculated from Kruskal-Wallis test followed by Steel-Dwass post-hoc test for **b** and **e**; *p < 0.05, **p < 0.01, ***p < 0.001). Data was obtained from three independent experiments. White bars in the images indicate 50 µm

Figure 4(a–a’’) are a representative 3D image of cell nuclei in the spheroid reconstructed by Imaris. As shown in Fig. 4(b), the nuclear volume increased significantly at day 7 and 14 compared to day 2 (*p* < 0.001 for day 2 vs day 7 and day 2 vs day 14). Figure 4(c–c’’) represent the nuclear volume depending on the nuclear position from the spheroid's center. A weak positive correlation was observed in the spheroids at day 2 (*r* = 0.095, *p* < 0.05) as well as other replicates shown in Fig. S2(a, a’’’). Spheroids at day 7 did not show a consistent trend between the samples (Fig. 4(c’) and S2(a’, a’’’’)). Spheroid at day 14 exhibited no correlation (*r* = 0.015, *p* = 0.86) between the position and nuclear volume, and similar trends were obtained in other replicates as represented in Fig. S2(a”, a’’’’’).

We also conducted a 3D image analysis to represent the sphericity for each nucleus in the spheroid as shown in Fig. 4(d–d’) while all the sphericity measurement results are also plotted in Fig. 4(e). The nuclear sphericity tended to increase as the incubation time increased, and significant differences were confirmed between spheroids at day 2, 7, and 14 (*p* < 0.01 for day 2 vs day7; *p* < 0.001 for day 2 vs day 14; and *p* < 0.001 for day 7 vs day 14). In Fig. 4(f–f’’), the relationship between the nuclear position from the center of the spheroid and the sphericity was plotted. The plots showed different manners among the replicates at day 2, 7, and 14, respectively, and no common trend was observed (Figs. 4(f–f’) and S2(b–b’’’’’)). However, the nuclei located at the surface of spheroid tended to be less spherical than those located in the inner spheroid. Altogether, we found out that the nuclei became hypertrophied and spherical over the 14-day culture.

## The ratio of chromatin condensation volume decreased as the cell nuclear size increased

**Fig. 5:**
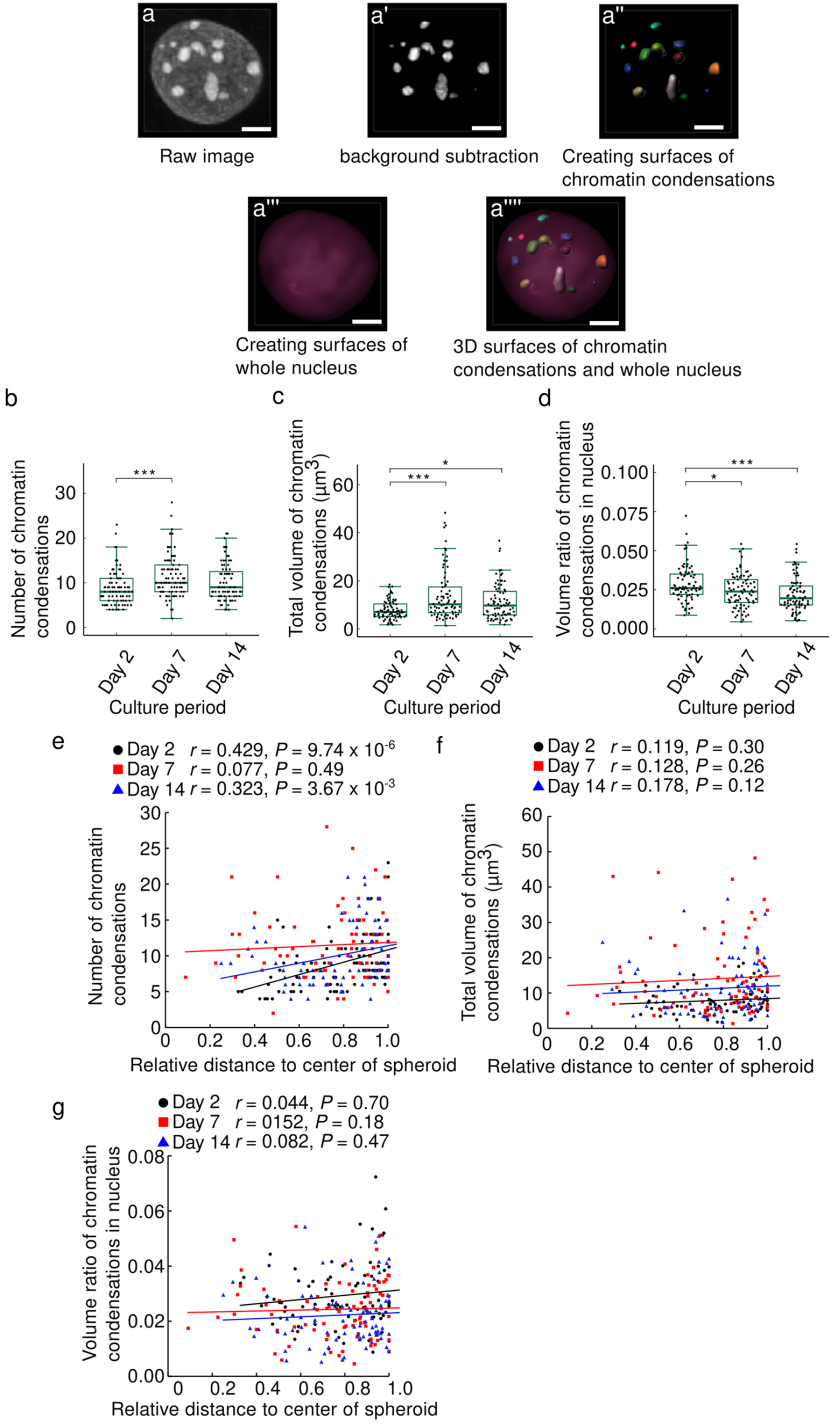
Three-dimensional spatiotemporal image analysis for chromatin condensations in nucleus in the ATDC5 spheroids at day 2, 7, and 14. **a**–**a’’’’** Overview of fabrication method for 3D surface of chromatin condensations in nucleus. White bars indicate 2 µm. **b** Boxplot of the number of chromatin condensations for the nucleus in spheroids at day 2, 7, and 14. **c** Boxplot of the total volume of chromatin condensations for the nucleus in spheroids on day 2, 7, and 14. **d** Boxplot of the volume ratio of chromatin condensations for the nucleus in the spheroids at day 2, 7, and 14 (p value was calculated from Kruskal-Wallis test followed by Steel-Dwass post-hoc test for **b**, **c**, and **d**; *p < 0.05, **p < 0.01, ***p < 0.001). **e** Relationship between the relative nuclear position from the center of the spheroid and the number of chromatin condensations in each nucleus at day 2, 7, and 14 respectively. **f** Relationship between the relative nuclear position from the center of the spheroid and the total volume of chromatin condensations at day 2, 7, and 14 respectively. **g** Relationship between the relative nuclear position from the center of the spheroid and the volume ratio of chromatin condensations in the nucleus at day 2, 7, and 14 respectively. The cell nuclei were randomly selected from each spheroid sample for analysis. The correlation between the nuclear position and the nuclear volume was examined from Spearman’s correlation test for **e**, **f**, and **g** (n, number of nuclei; N = 77 for day 2; n = 80 for day 7; n = 79 for day 14). Data was obtained from three independent experiments

As shown in Fig. 5(a–a’’’’), nuclei with distinct chromatin condensations were used to segment chromatin condensations for 3D image analysis. Figure 5(b) shows that the number of chromatin condensations in each cell nucleus was slightly larger at day 7 than day 2 (*p* < 0.001). Figure 5(c) represents that the total volume of chromatin condensations in each cell nucleus became significantly greater at the day 7 and 14 than day 2 (*p* < 0.001 for day 2 vs day 7; *p* < 0.05 for day 2 vs day 14). Furthermore, the ratio of chromatin condensation volume in each nucleus was significantly decreased at day 7 and 14 compared to day 2 (*p* < 0.05 for day 2 vs day 7; *p* < 0.001 for day 2 vs day 14) as shown in Fig. 5(d).

We further investigated the parameters examined in Fig. 5(b)–(d) (number, total volume, and volume ratio in each nucleus) depending on the nuclear position from the center of the spheroid. Figure 5(e) shows a significant positive correlation between the number of chromatin condensations and position at day 2 and 14, but not at day 7. The total volume and volume ratio of chromatin condensations depending on the relative nuclear position were also examined in Fig. 5(f) and (g), respectively. In both cases, there was no significant correlation in all groups.

## Discussion

In this study, morphological changes of chondrogenic progenitor cell-derived spheroids were quantitatively assessed through multi-scale spatiotemporal analysis, encompassing the actin cortex, nucleus, and chromatin condensation. Our present method captured the dynamic volume changes in chromatin condensations within chondrocytes throughout the course of early hypertrophic differentiation. This study implied the structural reorganization of nucleus during hypertrophic chondrocyte differentiation, which could not be investigated by conventional ex vivo, cartilage pellet or, and 2D in vitro models due to the technical limitations (Kimura et al. 2018; Kobayashi et al. 2016; Shukunami et al. 1997).

The ATDC5 spheroid model exhibited cellular and nuclear hypertrophy and increased sphericity as well as the up-regulation of the early hypertrophic chondrocyte differentiation marker genes (*Fgfr3* and *Col10a1*). In addition, the spheroid also exhibited the expression of COL10, which is a hallmark of hypertrophic chondrocytes (Linsenmayer et al. 1988). These results suggest that the spheroid culture of ATDC5 induced early hypertrophic chondrocyte differentiation, including morphological alterations mimicking characteristics of hypertrophic chondrocyte differentiation in vivo (Hunziker 1994; Kronenberg 2003; Prein et al. 2016). Within the day 2 spheroid, COL10 was expressed at the surface part of the spheroid where relatively large nuclei were localized. In addition, COL10 was detected throughout the whole area of the spheroid where hypertrophied nuclei resided within the day 7 and 14 spheroids. This correlative manner between COL10 production and nuclear volume is consistent with the histological feature observed in the hypertrophic zone within the epiphyseal plate in vivo (Kimura et al. 2018; Kobayashi et al. 2016).

The progression of hypertrophic chondrocyte differentiation within the spheroid (about 100 – 200 µm in diameter) model in this study, as well as the pellet model (about several millimeters in diameter) reported by Pretemer et al*.* (Pretemer et al. 2021), tends to initiate from the surface of the spherical structure and later at the inside. The typical shape of chondrocytes is known to become round during the event of differentiation from prechondrogenic cells to hypertrophic chondrocytes. However, the ATDC5 spheroid showed that the circularity of the cells and sphericity of nuclei were relatively smaller at the surface part where COL10 localized at day 2. In this study, type II collagen (COL2), a representative chondrocyte marker, was distributed in the inner side of the spheroid at day 2, whereas type X collagen (COL10) was distributed specifically at the outer edge (surface) of the spheroid (Kim et al. 2022). These results altogether may suggest that the microenvironment surrounding individual cells is different between the inside and outside of the spheroid at day 2, which in turn granted the cells different phenotypic features. It is generally believed that concentration gradients of nutrients and oxygen (Hirschhaeuser et al. 2010) and internal mechanical stresses exist in the spheroid (Boot et al. 2021; Dolega et al. 2017). The spatiotemporal changes in those properties over the 14-day culture period may have affected the spatial pattern of cellular and nuclear morphology within the spheroid.

The quantification of the volume of chromatin aggregates in individual cell nuclei revealed that the number and the volume of heterochromatin changed over 14 days of culture. Our system can analyze images of individual chromatin condensations in spheroid, whereas structural analysis of chromatin aggregates in cell nuclei has been limited to 2D cultured cells (Ghosh et al. 2022; Irianto et al. 2014). Compared to 2-day spheroids, the total chromatin condensation volume increased in 7- and 14-day spheroids, and the ratio of the chromatin condensation volume to the total cell nucleus decreased. This may suggest that the condensation state of chromatin condensations changed in concomitantly with the enlargement of the cell nucleus. One hypothesis is that the reorganization of the chromatin condensations was driven by the alteration in mechanical stress. In a previous study, it was reported that the nuclei of compressed fibroblasts have a large proportion of heterochromatin, and upon releasing the compression, the proportion of heterochromatin decreased to the pre-compression level (Damodaran et al. 2018). Similarly in the spheroids at day 2, individual cell nuclei are likely to be under greater compression and heterochromatin in a high compaction state due to the relatively higher expression of the RhoA/Rock than in the later period of culture. Previous studies on ATDC5 showed that the expression of RhoA/Rock signaling which regulates actin cytoskeleton formation is greater in undifferentiated chondrocytes than in differentiated chondrocytes (Wang et al. 2004; Woods and Beier 2006). On the other hand, from day 2 to day 7, the cell death may have reduced the cell density in the spheroid, and the ratio of heterochromatin might have decreased due to the relaxation of internal compressive stress. Although there was no significant correlation between the relative distance of each cell nucleus from the spheroid center and the total volume and volume ratio of chromatin condensations in the nucleus, the number of chromatin condensations was positively correlated only at day 2 and 14. Considering that the number of chromatin condensations increased from day 2 to day 7, it is certain that chromatin aggregates are dynamically reorganized in the spheroids. The mechanism and the causation between the reorganization of chromatin condensations and chondrogenic differentiation need to be clarified in future work. Taken together, the change in the physiological properties within the spheroid microenvironment caused by the cell death may play a role in triggering dynamic reorganization of the nuclear structure which induces epigenetic modification and alteration in the phenotypic features. To our knowledge, such a dynamic alteration in the nuclear morphology has not been addressed in any previous studies of 2D cartilage nodule model (Atsumi et al. 1990) or 3D cartilage pellet models (Chen et al. 2019; Pretemer et al. 2021).

It has been reported that hypertrophic chondrocytes undergo apoptosis during long bone development (Roach et al. 2004, 1995), and that cell membrane fragments of dead cells serve as materials for calcification (Hara et al. 2022, 2018). In our model, we confirmed that the spheroid culture induced severe cell death on day 2 in Fig. S1, followed by the hypertrophic chondrocyte differentiation on day 14. As shown in Fig. 3(f), the decrease in cell circularity from day 2 to day 7 can be attributed to the reduction in cell number inside the spheroid due to cell death, resulting in a relative increase in the proportion of elongated cells at the spheroid periphery. Further study will be required to verify whether there is a biological association between cell death and hypertrophic chondrocyte differentiation under the spheroid culture. Nevertheless, the present study showed the potential to indicate that the spheroid culture for chondrogenic progenitor ATDC5 cells mimics the initial stage of endochondral ossification. Combined with the differentiation induction chemical supplement (Atsumi et al. 1990; Temu et al. 2010; Yao et al. 2014) and the angiogenesis model (Zhao et al. 2021), which were not introduced in our system, it is expected to recapitulate late-stage hypertrophic chondrocyte differentiation leading to a 3D in vitro model of calcification to mimic further endochondral ossification process.

## Conclusion

In conclusion, this study presented the morphological alterations of nuclei and chromatin condensation during the early hypertrophic chondrocyte differentiation in the ATDC5 spheroid model. Our image analysis revealed the correlative manner of the morphological change in the nucleus with the progression of early hypertrophic chondrocyte differentiation. Dynamic reorganization of chromatin structure may play a role in determining cell fate and function during chondrogenesis.

## Supplementary Information

Below is the link to the electronic supplementary material.

Supplementary file1 (DOCX 4119 KB)

Supplementary file2 (DOCX 173 KB)

## Data Availability

The data that support the findings of this study are available from the corresponding author upon reasonable request.
