# Supplementary material for: Spatiotemporal analysis of multi-scale cell structure in spheroid culture reveals hypertrophic chondrocyte differentiation
Source: Cell Tissue Res. 2024 Jul 23;397(3):263–74. doi: 10.1007/s00441-024-03905-7 (PMC11371864; doi:10.1007/s00441-024-03905-7)

**Supplementary material**

**Fig. S1** Live-dead staining for spheroid at (a) day 2 and (b) day 14. Live (green) and dead (red) cells were stained with Calcein-AM and Propidium Iodide, respectively. White bars in the images indicate 50 µm.


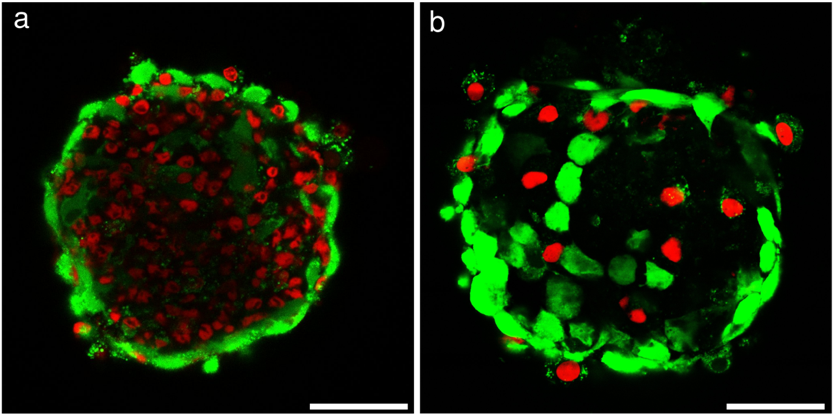


**Fig. S2**  Three-dimensional (3D) spatiotemporal image analysis for nuclear morphologies in spheroids at day 2, 7, and 14. Results of the other two samples (#2: (a) day 2, (a’) day 7, (a’’) day 14; #3: (a’’’) day 2, (a’’’’) day 7, (a’’’’’) day 14) from two independent experiments for the correlation analysis between the nuclear position from the center of the spheroid and nuclear volume at day 2, 7, and 14. (b) The correlation analysis between the nuclear position from the center of the spheroid and nuclear sphericity at day 2, 7, and 14. Results of two samples (#2: (b) day 2, (b’) day 7, (b’’) day 14; #3: (b’’’) day 2, (b’’’’) day 7, (b’’’’’) day 14) from two independent experiments. The correlation between the nuclear position and the nuclear volume or sphericity was examined by Spearman’s correlation test (*n*, number of nuclei).


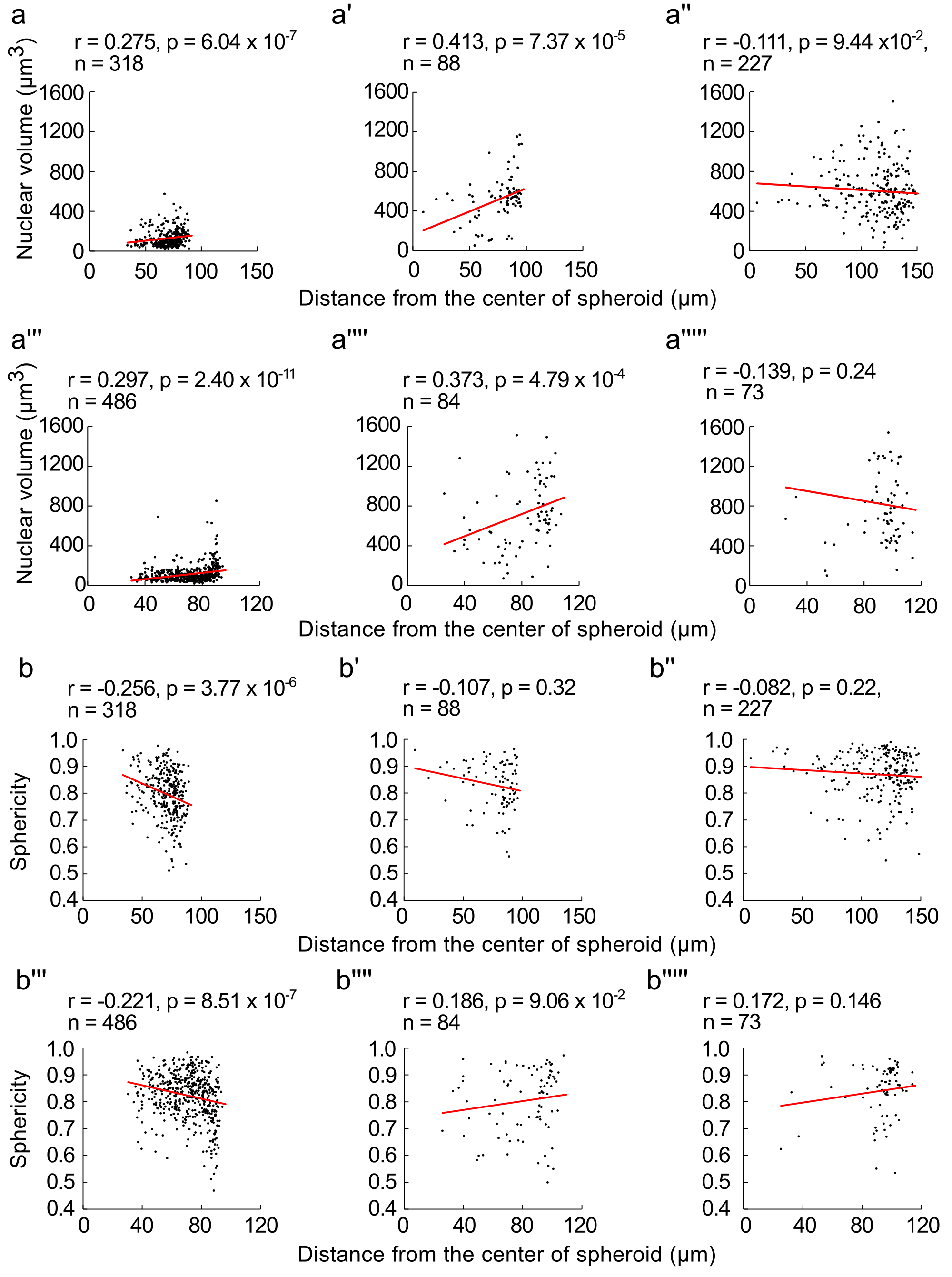

Supplement: Supplementary file 1 — Supplementary file1 (DOCX 4119 KB) [file 441_2024_3905_MOESM1_ESM.docx]
