# Supplementary material for: Spatiotemporal analysis of multi-scale cell structure in spheroid culture reveals hypertrophic chondrocyte differentiation
Source: Cell Tissue Res. 2024 Jul 23;397(3):263–74. doi: 10.1007/s00441-024-03905-7 (PMC11371864; doi:10.1007/s00441-024-03905-7)
Supplement: Supplementary file 2 — Supplementary file2 (DOCX 173 KB) [file 441_2024_3905_MOESM2_ESM.docx]

Table S1. Designs of primers.

| **Gene** | **Forward primer** | **Reverse primer** | **Amplicon size (bp)** |
| --- | --- | --- | --- |
| ***Gapdh*** | TGTTCCTACCCCCAATGTGT | GGTCCTCAGTGTAGCCCAAG | 137 |
| ***Acan*** | CTGACATTTGAGGAGGCACA | GGCTCACAATGGGGTATCTG | 151 |
| ***Col2a1*** | GTGTGTGTGACACTGGGAATG | GTTCTCCTTTCTGCCCCTTT | 159 |
| ***Fgfr3*** | CACCGACAAGGAGCTAGAGG | CTGCCAGCCTCATCAGTTTC | 165 |
| ***Col10a1*** | GCAGAGGAAGCCAGGAAAG | CTCTTTATGGCGTATGGGATG | 209 |
| ***Mmp13*** | CCTAAGCATCCCAAAACACC | AACATAAGGTCACGGGATGG | 218 |
